# Label-Free, High Resolution, Multi-Modal Light Microscopy for Discrimination of Live Stem Cell Differentiation Status

**DOI:** 10.1038/s41598-017-18714-y

**Published:** 2018-01-15

**Authors:** Jing Zhang, Emilia Moradi, Michael G. Somekh, Melissa L. Mather

**Affiliations:** 1 Institute for Science and Technology in Medicine, Keele University, Stoke-on-Trent, ST4 7QB United Kingdom; 2 Optics and Photonics, Faculty of Engineering, University of Nottingham, Nottingham, NG7 2RD United Kingdom; 3 Department of Electronic and Information Engineering, The Hong Kong Polytechnic University, Hung Hom, Hong Kong

## Abstract

A label-free microscopy method for assessing the differentiation status of stem cells is presented with potential application for characterization of therapeutic stem cell populations. The microscopy system is capable of characterizing live cells based on the use of evanescent wave microscopy and quantitative phase contrast (QPC) microscopy. The capability of the microscopy system is demonstrated by studying the differentiation of live immortalised neonatal mouse neural stem cells over a 15 day time course. Metrics extracted from microscope images are assessed and images compared with results from endpoint immuno-staining studies to illustrate the system’s performance. Results demonstrate the potential of the microscopy system as a valuable tool for cell biologists to readily identify the differentiation status of unlabelled live cells.

## Introduction

Methodologies to assess the purity of therapeutic stem cell populations and differentiation status of cells during *in vitro* culture are urgently needed. Technologies to address this need will enable optimisation of culture protocols, aid in reducing the risk of implanting proliferating tumour forming cells, facilitate maintenance of a stable cell phenotype during *in vitro* expansion and ultimately improve the efficacy of current and emerging stem cell therapies^1–3^. There are a number of existing cellular and molecular assays that are being used to characterise cell populations *in vitro*, the majority of which are experimentally intensive, invasive and often destructive rendering time-course experiments impossible. These methods include flow cytometry^4^, which requires cells to be harvested in order to have a single cell suspension, immuno-staining^5^, which either requires cells to be fixed if the marker of interest is inside the cell or the use of cell surface markers, which may result in a change in cell morphology, and molecular methods (e.g. reverse transcription-polymerase chain reactions) which are destructive and typically provide information on a population level not at a cell-by-cell level^6^.

An alternative approach to the above assays is fluorescence microscopy which uses fluorescent markers to provide high resolution information on the distribution and dynamics of different biomolecules in cells^7^. Notably, the differentiation status of live stem cells can be assessed using fluorescence microscopy to detect lineage-specific cell surface markers by fluorescently labelled antibodies^8,9^ and transfection of cells with fluorescent proteins^10,11^. A major drawback of these approaches is the necessary modification of cells, either via incorporation of labels or gene transfection, and the potential perturbation to cells caused by such modification^12^. Moreover, long term monitoring of cells with fluorescence microscopy can cause photo-toxicity to cells and most importantly the use of exogenous labels or gene transfection render cells unsuitable for subsequent clinical implementation.

There is thus a requirement for a non-invasive, label-free approach to discriminate distinct differentiation stages of therapeutic stem cells and to identify their differentiation potential, viability and proliferation in culture. Advanced label-free light microscopy techniques are prime candidates to meet this requirement as they can be applied rapidly on both a single cell and population level, provide high resolution images and are amenable to long term time-lapsed studies of live cells. To this end success has been found through the combination of non-linear optical microscopy techniques such as coherent anti-Stokes Raman scattering (CARS) and second harmonic generation (SHG) which have been shown to provide similar sensitivity as destructive biochemical methods for the analysis of stem cell differentiation, with the added benefit of providing structural microscopic information^13^. These techniques certainly will play a role in the non-destructive and label-free analysis of stem cells, however, their implementation is far from straightforward and have limited throughput due to their reliance on laser scanning and low light levels to form images. Thus, this approach does not satisfy the need for quick, sensitive screening methods to characterise stem cells particularly for therapeutic use in a timely manner^14^.

Full field microscopy techniques that can be easily integrated into microscopy systems commonly found in biological laboratories are thus desirable. In this paper a novel multi-modal light microscope for non-invasive, label-free characterisation of live cells based on the use of evanescent wave microscopy and quantitative phase contrast (QPC) microscopy is presented. Importantly the metrics obtained from the two imaging modalities are used in combination which greatly enhances the utility and the power of the technique. Further, the experimental approach used here overcomes limitations of previously reported methods^15^ by exploiting evanescent wave illumination to produce high-contrast images of a thin optical section above a cell culture substrate combined with quantitative phase contrast microscopy that enables arbitrary contrast to be obtained (e.g. bright field transmission and dark field). To assess the capability of the microscopy system presented the differentiation of live immortalised neonatal mouse neural stem cells (C17.2)^16,17^ is studied over a 15-day time course. Metrics which combine measures from microscope images obtained from both modalities are compared with results from endpoint immuno-staining studies to illustrate the performance of the microscopy system presented to evaluate stem cell differentiation status in comparison to currently used fluorescent microscopy techniques.

## Results

### Principle and design of microscopy system

Here the proximity of the cell membrane is imaged using total internal reflection microscopy (TIRM) which provides excellent contrast, monotonic response over large distances and excellent compatibility with conventional unmodified high numerical aperture objectives^18^. TIRM utilizes evanescent field illumination which is generated when light travelling in a material is incident onto a material with a lower refractive index at a sufficiently high angle to cause total internal reflection. The evanescent waves generated decay rapidly away from the coverslip with a penetration depth of the order of hundreds of nanometres. Contrast in TIRM images arises from differences in the refractive index of material above the coverslip that either fulfil the conditions for total internal reflection or frustrate these conditions. The incident angle is chosen such that regions corresponding to the aqueous media in the field of view will appear as bright areas in the image. In the case of cells the refractive index is typically higher than that of the surrounding solution and as such the condition for total internal reflection is frustrated and light is coupled out of the illumination beam. As a result cells appear in the image as darker regions against a bright background. In this work evanescent illumination was achieved through the use of an incoherent light source (Light Emitting Diode (LED)) that is spatially filtered using an annular mask and then re-imaged into the back focal plane of a high numerical aperture objective lens.

QPC imaging is performed using a phase spatial light modulator (SLM) to obtain a set of images at equal phase steps from a reference channel. In practice, an image of a ring is displayed on the SLM as a surrogate phase plate with the phase being controlled by the drive signal to the SLM. The optical design chosen enables the full numerical aperture (NA) of the optics to be utilised and the amplitude and phase of the image field to be acquired, which can subsequently be post-processed to enable arbitrary imaging modes such as bright field transmission and dark field to be acquired. Finally, unlike other systems reported in the literature the multi-modal microscope developed here is designed such that images with different sized fields of view can be obtained simultaneously enabling the study of single cells (field of view 130 μm) and cell populations (field of view 400 μm). Advantageously the image area achieved for cell population studies is 2.56 times greater than the designed field of view of 250 μm and images obtained display no significant degradation in resolution.

### Live cell imaging

**Figure 1:**
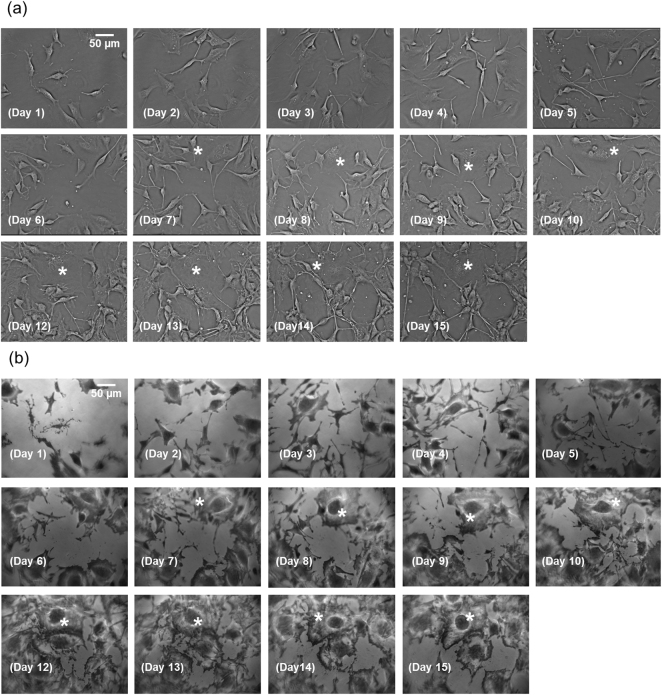
(**a**) Phase contrast images obtained over the time course of culture. The day of culture each image was obtained on is shown. The scale bar for all images is printed on the image obtained on Day 1. For the purposes of comparison with corresponding cells in the TIRM images selected cells are marked (*). (**b**) TIRM images obtained over the time course of culture. The day of culture each image was obtained on is shown. The scale bar for all images is printed on the image obtained on Day 1. For the purposes of comparison with corresponding cells in the phase contrast images selected cells are marked (*).

To illustrate the performance of the microscope a well-characterized, stable, clonal population of mouse neural stem cells (C17.2)^16,17^ was imaged over 15 days, a timeframe known to be an active period of cell differentiation^17,19^. Images obtained using TIRM and QPC microscopy modes taken at the same time point and field of view are shown in Fig. 1. There is a clear difference in the appearance of images obtained with the two modalities which can be related to the extent to which light interacts with the sample. The QPC images are based on transmitted light that has interacted with the full depth of cells in the culture dish while the TIRM images only show cellular components that are within the evanescent field (~100 nm to 200 nm) emerging from the cell culture substrate. As a result, cells that are not in the proximity of the substrate are not seen in the TIRM images. The contrast in the QPC images arises from differences in optical path length within the sample. In the case of flat cells adhered to the surface, the contrast is poor using QPC imaging, however they are seen in excellent contrast with TIRM. To illustrate this point an asterisk has been added in regions where this is particularly evident in the corresponding TIRM and QPC images in Fig. 1.

Over the 15 day time course the differences in images obtained from the two modalities increases. From the literature, it is known that after 5 days in culture a mixed cell population starts to form as cells begin to differentiate^19^. This time point corresponds well to the onset of notable differences in the observable features of cells between the images obtained with QPC and TIRM and is particularly apparent from day 10 onwards. Notably at the end of the time course networked colonies of cells are seen in the QPC images, typical of a neural phenotype, while the TIRM images depict large, flat cells typical of an astrocyte phenotype, these are obscured in the QPC images by overlaying cells. The morphological differences and complementary information obtained from TIRM and QPC images provides support for the hypothesis that cell differentiation status and hence phenotype can be studied via the multi-modal microscopy system presented here.

### Multi-modal imaging

**Figure 2:**
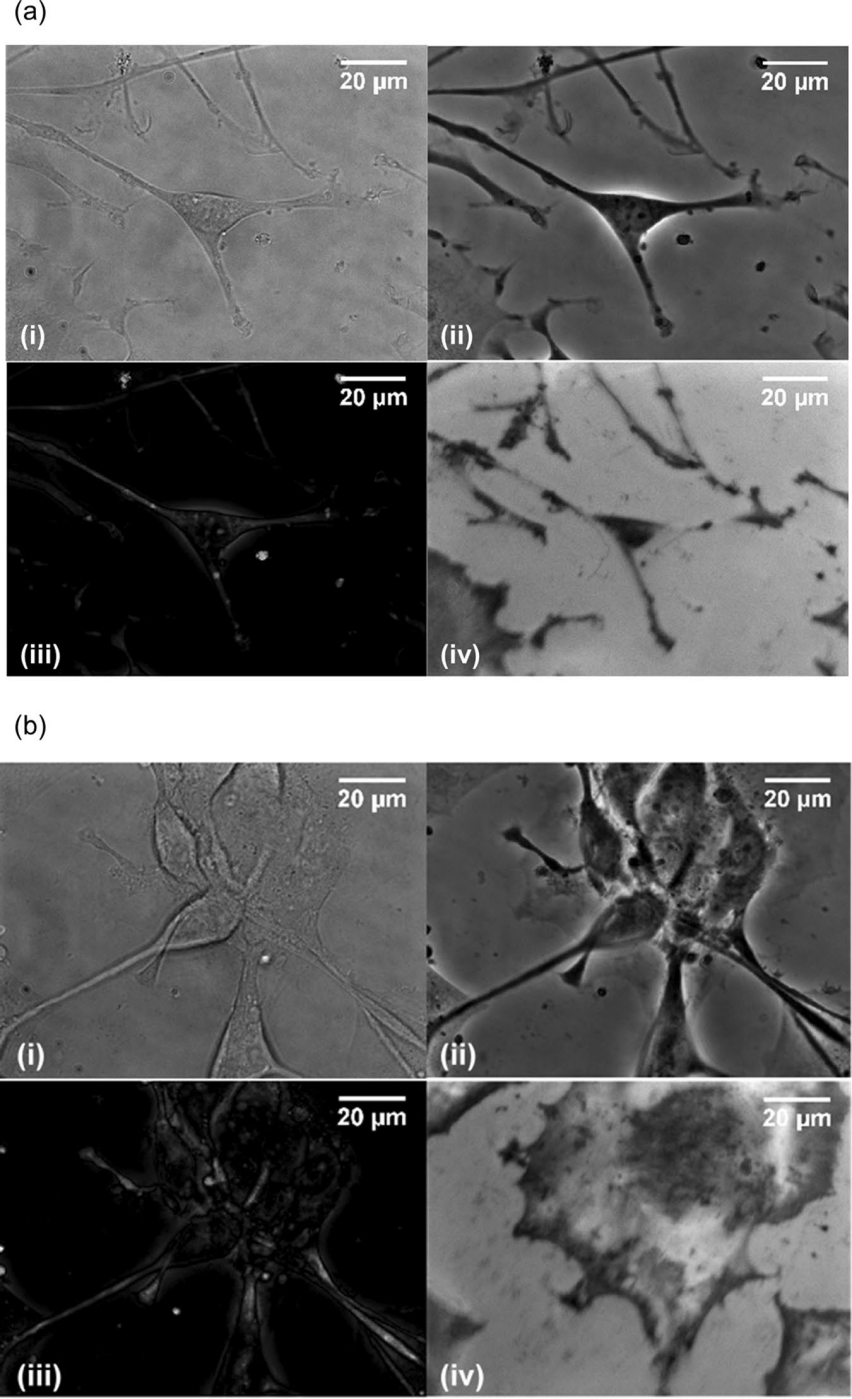
(**a**) Multi-modal images obtained on day 5 of culture: (i) Bright field transmission; (ii) QPC; (iii) dark field; (iv) TIRM. (**b**) Multi-modal images obtained on day 15 of culture: (i) Bright field transmission; (ii) QPC; (iii) dark field; (iv) TIRM.

Figure 2 shows bright field transmission, phase contrast, dark field and TIRM images obtained using the small field of view (FOV) for inspection of cells on day 5 (Fig. 2a) and day 15 (Fig. 2b). The difference in the images obtained with each of the modalities is clear. The morphology of cells seen in the transmission and phase contrast images is comparable; however, the contrast in the transmission image is poor owing to the low attenuation of light by the cells. Strong contrast between the cells and their surroundings is obtained in the phase contrast images which is a result of interference between the zero order and diffracted order of light through the cellular material. The contrast seen in the dark field images arises from light scattered by the cells which here is useful to highlight raised features. As mentioned earlier contrast in images obtained using TIRM is related to the separation of the sample to the surface of the cell culture substrate.

The similarity of the cell morphology depicted in all images obtained on day 5 suggests cellular material is in close proximity to the substrate and the area studied consists predominantly of one cell type. Results from day 15 are in stark contrast showing clear differences between the content and morphology of cells contained in the TIRM image and the corresponding bright field transmission, phase contrast and dark field images. Two distinct cell morphologies are observed on day 15 which appear to be anchored to one another in a layered manner. The top layer is typified by colonies of cells with small bodies connected via thin projections that form networks. This morphology is indicative of neurons which are known to form networks near day 10^17^. The bottom layer shows flatter rounded cells with a much larger cell body indicative of astrocytes.

**Figure 3:**
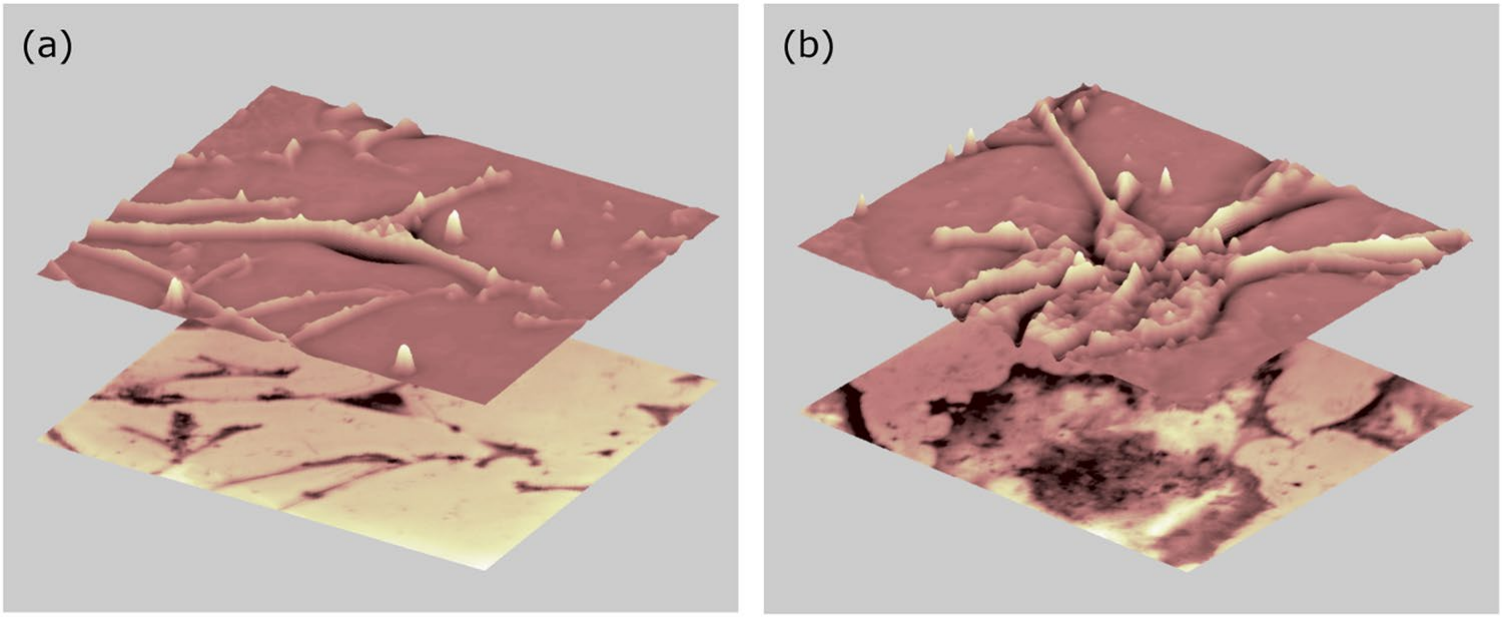
3D topography of cells overlayed on TIRM images from day 5 (**a**) and day 15 (**b**).

Further information can be obtained by reconstructing the 3D morphology of cells using the quantitative phase contrast capability of the multi-modal microscope. Here the optical phase is extracted by acquiring a set of phase contrast images with a π/2 phase step in the reference channel described earlier. Figure 3 displays the reconstructed topography of cells shown in Fig. 2 overlaid on the corresponding TIRM image. The overlay of images acquired on day 5 (Fig. 3a) shows good correspondence between cells detected in the upper and lower images. A central dark region can be seen in the cell at the middle of the TIRM image in a region corresponding to the location of the cell nucleus. At the same position in the image of cell topography a dip in the cell height is seen compared to the surrounding cytoplasma. On day 15 (Fig. 3b) the TIRM and topography images are significantly different. First the anchorage of the top layer of neurons to the bottom layer of cells is apparent due to their lateral co-localisation. Second, the thickness of the cells in the two images is distinct with the TIRM image displaying a rounded flat cell, indicative of astrocytes, and the upper image depicting thicker neurons. Overall, the results shown in Figs 2 and 3 highlight the utility of multi-modal imaging to track cell features known qualitatively to be related to phenotype.

### Metrics for discrimination of differentiation status

To quantitatively assess the differentiation status of cells and to develop a protocol for discrimination of cell type, non-overlapping cells in all images obtained at each time point were identified and analysed via image processing (as described in the online methods). Briefly, images were inspected visually to identify regions of interest containing non-overlapping cells. Multiple cropped versions of each image were created corresponding to identified regions of interest. The perimeter of individual cells in each cropped image were manually traced to aid subsequent automated image processing. Image processing involved the use of Matlab (The Mathworks) in-built functions to produce binary images corresponding to each individually segmented cell. These binary images were then quantitatively analysed to calculate individual cell areas and aspect ratios, which denotes the ratio of the major axis length to the minor axis length of the cell, for multiple cells in TIRM and QPC images. These metrics are known from the literature to aid morphological assessment of cell phenotype with the cell area being of importance as progenitor cells can be identified from astrocytes due to the smaller size of progenitors. Also, the aspect ratio provides a measure of the relationship between the cell body diameter and the cell length, which can aid discrimination between the elongated morphology of progenitor cells and neurons from that of astrocytes. For clarity, the number of individual cells analysed on each day of culture as well as the median values of the area and aspect ratio for each modality are tabulated in the Supplementary Information.

**Figure 4:**
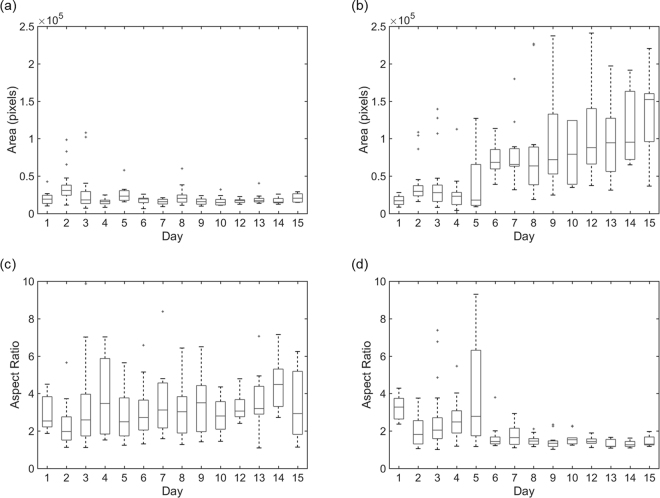
Boxplots, showing the area and aspect ratios calculated from the QPC and TIRM images over the 15 day time course, were created to assess the differentiation status of cells from image metrics on a population level. (**a**) QPC Area; (**b**) TIRM Area; (**c**) QPC Aspect Ratio; (**d**) TIRM Aspect Ratio. In the boxplots, the boundary of the box closest to zero indicates the 25th percentile, the line within the box marks the median, and the boundary of the box farthest from zero indicates the 75th percentile. Whiskers above and below the box include points that are not outliers. Details of the number of cells analysed at each day of culture are shown in the Supplementary Information.

The results for all cells analysed at each time point as described above were combined to provide information on a population level and shown using box plots to enable assessment of the spread and skewness of the results, as shown in Fig. 4. For each box, the central mark is the median of the cell population analysed at each time point, the edges of the box are the 25th and 75th percentiles, the vertical lines extending from the box (whiskers) expand to the most extreme data points not considered outliers, and outliers are plotted individually. Figure 4(a) and (b) display the area in pixels, spread of values at each time point and variation in results over the 15 day time course for the QPC and TIRM images. Similar values for area are attained on days 1, 2, 3, 4 and 5 for the two modalities, while for later time points the areas extracted from the QPC images are consistently lower than those from the TIRM. It is hypothesised that the difference in area measurements indicates the onset of active differentiation with TIRM being particularly sensitive to the presence of astrocytes. As time progresses the variation between the results from QPC and TIRM images increases in line with the emergence of a mixed cell population, highlighting the sensitivities of QPC and TIRM to different cell types. This is most apparent on day 15 when the median area found from the TIRM images is greater than that in the QPC images by more than a factor of 3. The variance for each time point is also of note which is high for metrics extracted from the TIRM images, particularly at the later time points. This could be linked to the increased density of cells seen in Fig. 1(b) that affects the packing density and hence cell size.

Figure 4(c) and (d) show plots of the aspect ratio calculated from the QPC and TIRM images. The median aspect ratios calculated for the first 5 days of culture in both the QPC and TIRM images are comparable. A notable observation is the large variation in the aspect ratio of cells analysed on day 5 which may be indicative of rapidly changing cell morphology and hence onset of differentiation. At later time points the aspect ratio from the TIRM image decreases while no such trend is seen in the QPC images. This reduction in the aspect ratios calculated from the TIRM images suggests a change from an elongated cell morphology towards a circular morphology characteristic of differentiation of neural progenitor cells into astrocytes. Unlike the area results, however, the variance in aspect ratio for each time point and over the later time points, for results obtained from the TIRM images, is low indicating that although the overall size of astrocytes changes with time their circular morphology is quite constant. The median values for aspect ratio calculated from the QPC images over the 15 day time course are considerably greater than one indicating cells maintain an elongated morphology characteristic of both neural progenitor cells and neurons. Another noteworthy observation is the spread in results from day 6 onwards in the QPC images is visibly higher than in the TIRM images. This is of relevance for discrimination between different cell types particularly as the comparatively large spread in QPC results highlights the dynamic nature of neuron morphology as they probe their environment and form networks as compared to the more static behaviour of astrocytes once in proximity of the substrate.

**Figure 5:**
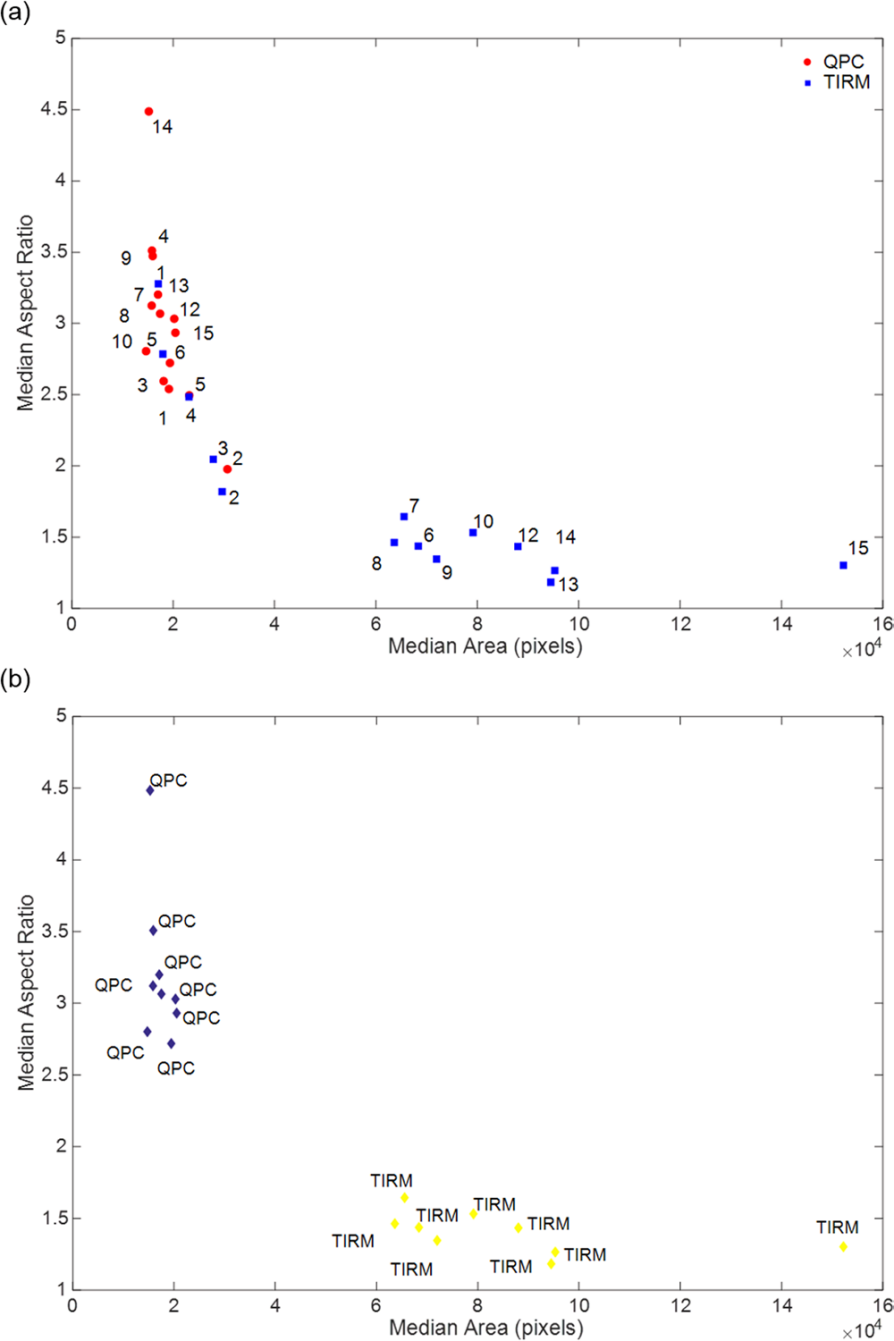
(**a**) Scatter plot showing median aspect ratio versus median area over the time course of cell culture to aid discrimination of cell type. TIRM results are displayed as solid squares and QPC results as solid circles. Each point is labelled with the day of culture the results correspond to. Results are grouped into two regions in the plot. One region contains data from both QPC and TIRM images and the other region contains data corresponding to results from TIRM images alone from day 6 onwards, corresponding to cell morphology indicative of astrocytes. (**b**) Scatter plot of results from both imaging modalities for days 6 to 15 of culture grouped by colour using k-means analysis. Points are labelled with the imaging modality they correspond to.

The results shown in Fig. 4 provide a basis for establishment of a protocol for discrimination between the different cell types using the median area and aspect ratio over time. To explore this, the median aspect ratio was plotted against the median area for both modalities over the time course of culture (Fig. 5(a)). The results shown in Fig. 5(a) occupy two regions of the plot with one region containing data from both QPC and TIRM images and another region containing data corresponding to results from TIRM images alone from day 6 onwards. The results contained in this second region correspond to large cells with low aspect ratio, indicative of astrocytes. This is a useful observation, however, such an approach in this case only enables identification of astrocytes.

As a supplementary measure, statistical analysis was applied to the area and aspect ratio data obtained from both imaging modalities at each time point. Specifically, a Wilcoxon rank sum test, which is a non-parametric analogue to the t-test, was chosen as it is well suited to analysis of small samples sizes and does not require the assumption that the data comes from a normally distributed data set. Here the Wilcoxon rank sum test was used to test the null hypothesis that the areas of cells calculated from QPC and TIRM images at each time point come from continuous distributions with equal medians. The Wilcoxon rank sum test was performed for the area results and also the aspect ratio results on the single set of data from QPC and TIRM images obtained on each day of culture. The results, tabulated in the Supplementary Information, show that for both area and aspect ratio results the null hypothesis is not rejected at a 5% significance level for results obtained on the first 5 days of culture. For all later time points the null hypothesis is not accepted. This division correlates with the time points cells are known to be in the progenitor state from those when they are known to commence differentiation. Thus, we suggest that the results of the Wilcoxon rank sum test are useful for separating cells in the progenitor state to differentiated cells.

Data obtained from day 6 onwards was then considered in isolation to other time points and subjected to k-means cluster analysis with a view to quantitatively discriminate between the hypothesised neurons and astrocytes, Fig. 5(b). K-means clustering is an unsupervised, iterative algorithm that assigns observations to one of k clusters as defined by centroid values. Each centroid is the mean of the points in that cluster and the squared Euclidean distance can be used to partition the data. The k-means cluster analysis was performed using an in-built command in Matlab based on the hypothesis that the data would fall into two groups, one representative of neurons and one of astrocytes. As seen in Fig. 5(b) the data does fall into two categories and of note is that the QPC and TIRM results are in separate clusters. Further, the values for area and aspect ratio for these clusters correspond to values characteristic of neurons, in the case of QPC results, and astrocytes, in the case of TIRM results.

The above analysis provides a potential protocol for discrimination of cell type in the case of the C17.2 cells studied here based on the combined use of the Wilcoxon rank sum test and k-means cluster analysis. We suggest that this approach would be applicable to other cell types that undergo distinct changes in morphology and adhesion during culture.

### Comparison to immuno-staining

**Figure 6:**
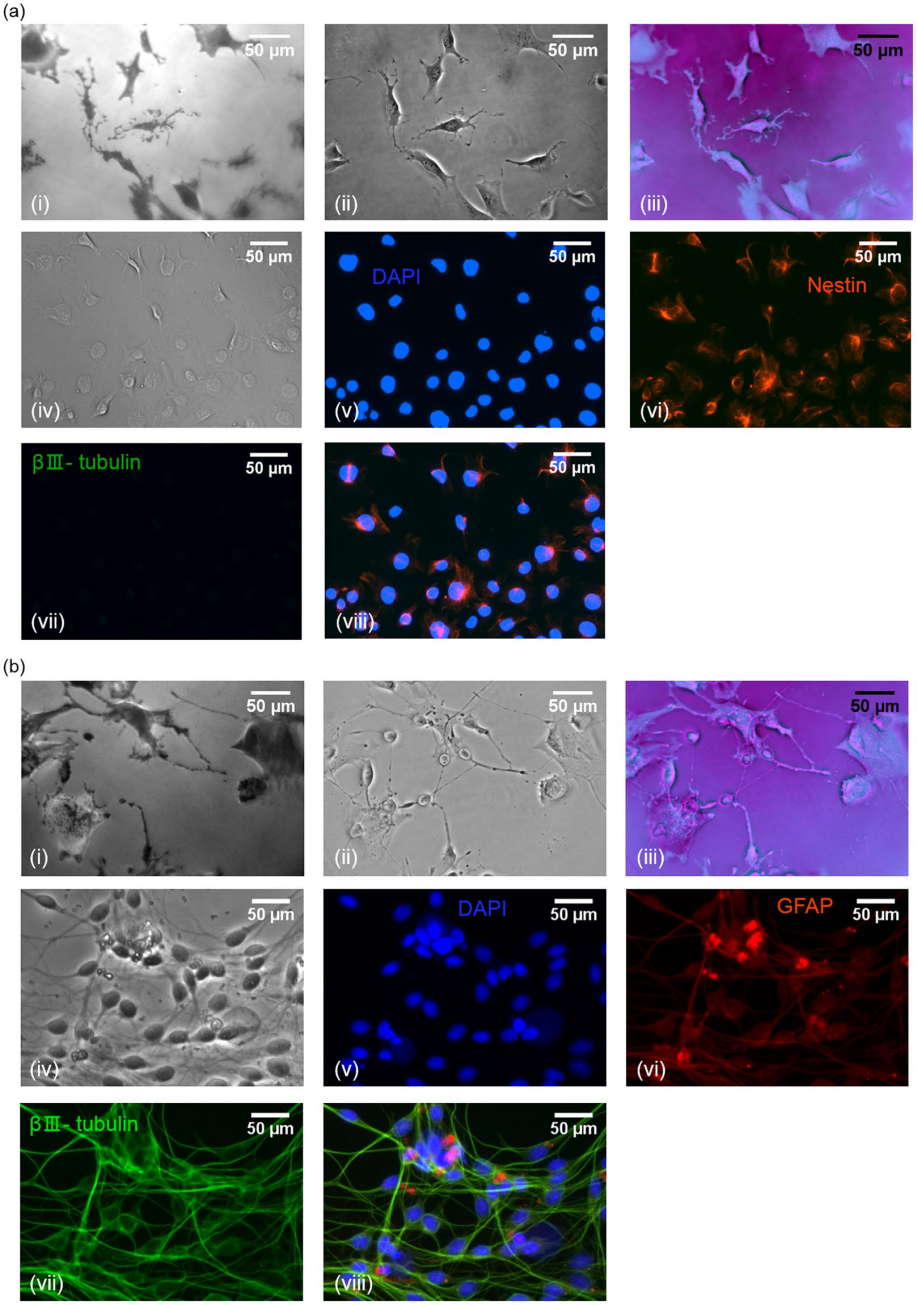
(**a**) Day 1: TIRM image (i), Phase image (ii), overlay of TIRM and Phase image (iii); Phase image (iv); DAPI staining showing cell nuclei (v); Representative image of immunofluorescence detection of nestin, biomarker for progenitor cells (vi); Representative image of immunofluorescence detection of βIII- tubulin, biomarker for neurons (vii); overlay of immunostained images. (**b**) Day 15: TIRM image (i), Phase image (ii), overlay of TIRM and Phase image (iii); Phase image (iv); DAPI staining showing cell nuclei (v); Immunofluorescence staining of GFAP representing astrocytes (vi); Immunostaining of βIII- tubulin neurons (vii); Overlay of immunostained images (viii). DAPI.

To investigate the hypothesis that the multi-modal microscopy system used here can assess changes in the cell type over time, multi-modal images of immuno-stained cells from the same cell population at the same time points were obtained and compared with phase contrast and fluorescence microscopy. Figure 6(a) shows a phase contrast image of cells fixed on day 1 of culture along with fluorescent images depicting the location of cell nuclei, reported via binding of DAPI stain to DNA, the presence of progenitor cells, identified through detection of the neural progenitor cell marker nestin (a type-IV intermediate filament identifying neural progenitor cells) and absence of neurons, investigated via immunostaining of βIII- tubulin. As a comparison QPC and TIRM images as well as a false coloured overlay of these images are shown. On a population level, cell morphology in the QPC and TIRM images is predominantly of one type suggesting the presence of a single cell population which corresponds to the findings from immuno-staining. Fluorescent images of immuno-stained cells from day 15 of culture (Fig. 6(b)) confirm the presence of neurons, via the expression of biomarker βIII-tubulin (part of the microtubular complex identifying neurons), and astrocytes, via the expression of specific biomarker glial fibrillary acidic protein (GFAP, a type-III intermediate filament identifying astrocytes). Visual inspection of cells on a population level imaged via QPC and TIRM on day 15 provides evidence of distinct cell morphologies typical of neurons and astrocytes, correlating well with the results from fluorescent images of immuno-stained cells. Future work is planned in which the label-free microscope is equipped with fluorescence imaging capabilities to enable direct comparison of the morphology of individual cells with the corresponding fluorescence reporters over the whole-time course of culture.

## Discussion

A new approach to label-free discrimination of live stem cell differentiation status has been presented using high resolution, multi-modal light microscopy. This approach harnesses the capability of TIRM to produce high contrast images of cellular components within the proximity of a substrate (~100 nm to 200 nm), providing critical information about cell adhesion, and a phase stepping approach to QPC imaging, enabling the 3D morphology of cells to be assessed. Here the multi-modal microscope has been applied to characterisation of the stages of neuronal cell differentiation over a 15 day time course. Images obtained highlight the complementary information available from TIRM and QPC imaging enabling the strengths of these techniques to be combined to provide a multi-layered, rich data set for analysis. Importantly simple metrics extracted from corresponding images obtained using both microscopy techniques were found to deviate in value from one another over the time course. It is argued that the methods and metrics identified in this work could form the basis of a protocol for quantitative discrimination of cell type in a rapid and label-free manner, which could be applied to a wide range of cells undergoing similar morphological changes as the C17.2 cells studied in this work. Moreover, the precision and throughput of morphological feature extraction and hence robustness of the methodology will be enhanced by the growing number of computational tools emerging for automated analysis of microscopy images^20^.

Overall the multi-modal microscope described in this work presents a novel and timely approach to address key challenges relating to the identification of the appropriate cell source for use in the clinic, acquisition of large amounts of high purity cells and maintenance of a stable cell phenotype during *in vitro* expansion. Further, the approach taken here could replace the often tedious aspect of stem cell research which is the need to characterise cells throughout culture, in a label-free manner. Importantly this technique provides cell biologists with the necessary tool and strategy to identify cells at early stages of differentiation enabling adjustment of culture conditions to alter the fate of cells and potentially improve the yield of clinically applicable cells.

## Methods

### Microscope

**Figure 7:**
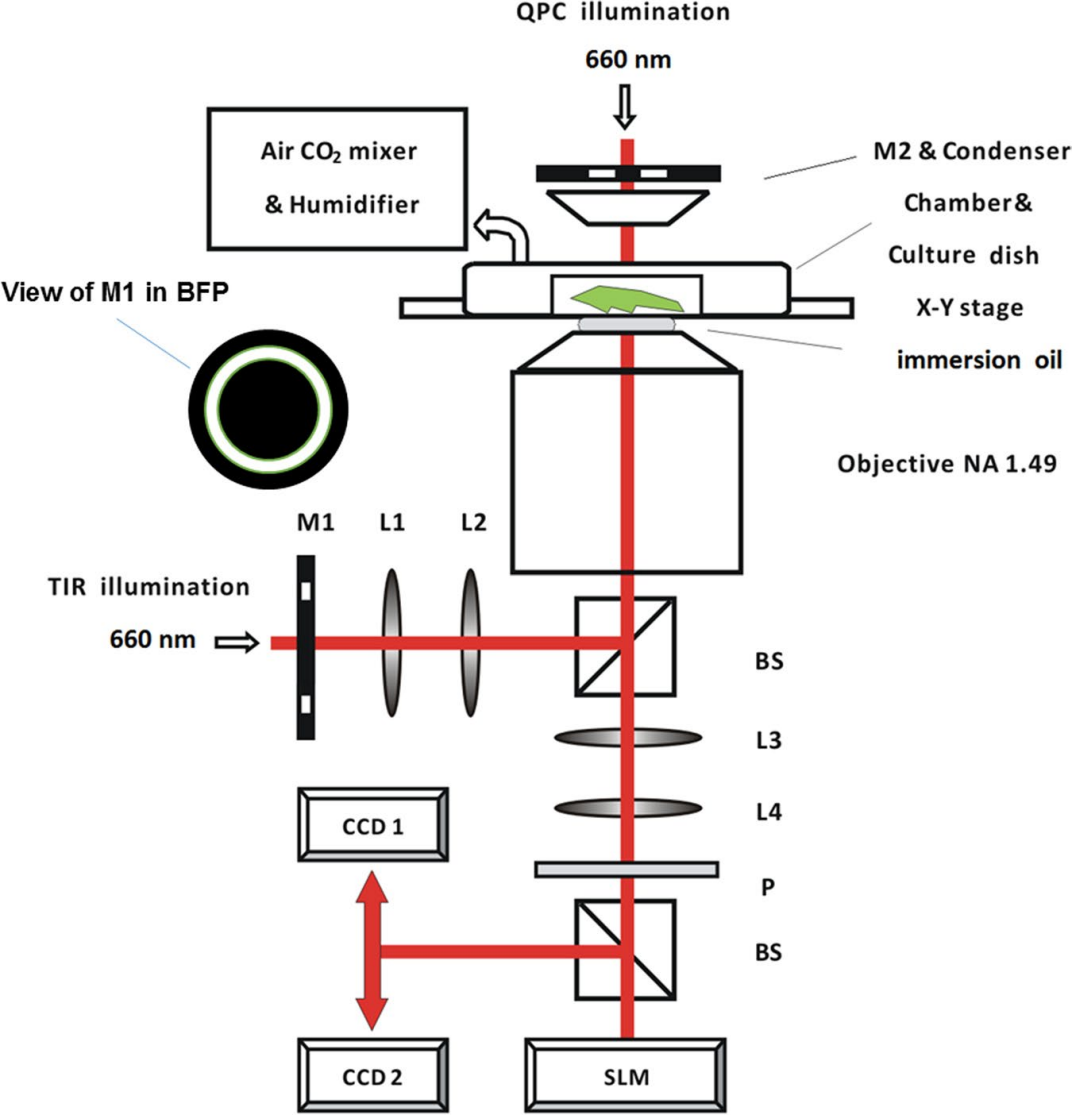
Schematic of optical system. Lens(L); beam splitter (BS); polariser (P); spatial light modulator (SLM); charged coupled device (CCD); mask (M1 & M2), back focal plane (BFP).

A schematic of the microscope set-up is shown in Fig. 7. Two light emitting diodes (LEDs) of the same wavelength (Thorlabs, super LED 660 nm) are used to illuminate the sample, one from the top for QPC imaging, and the other from the bottom of the cell culture dish for TIRM. A wavelength of 660 nm was chosen as long wavelength light is less photo-toxic than shorter wavelengths and thus enables live cells to be imaged for prolonged periods with a lower risk of adverse effects on cells. As both illumination sources have the same wavelength they are operated sequentially, although this produces a time delay of the order of several milliseconds between the different imaging modes, this is of no consequence in our study of the relatively slow process of cell differentiation. Additionally, the use of one wavelength obviates the need to correct corresponding images for chromatic aberration.

In terms of the optical components a high NA objective lens (Nikon NA1.49, × 60 CIF) forms the main component of the instrument. Such a high NA enables large illumination angles to be used which is necessary to produce evanescent wave illumination in the TIRM arm of the instrument. As shown in Fig. 7 the TIR illumination arm includes a mask located at the conjugate plane of the back focal plane (BFP) of the objective. This mask is used to pass angles of illumination only slightly greater than the critical angle between the coverslip and sample medium (typically over a range of 3° to 5°). This range of angles appears to give optimal TIR image contrast^18^.

A crucial element in the QPC imaging arm is the spatial light modulator (SLM, Hamamatsu × 10468–06), which is positioned at the conjugate plane of the BFP of the objective. The SLM allows phase patterns to be input digitally enabling fast and automatic interchange between arbitrary imaging modes without physically modifying the configuration of the optical system, effectively acting as a programmable phase plate in a conventional phase contrast microscope. The QPC illumination arm contains a long working distance objective lens (Mitutoyo NA 0.28, 10x) which functions as the condenser, and an annular ring located at a point corresponding to the conjugate of the back aperture of the condenser.

In order to obtain images with two different fields of view two charged coupled device (CCD) cameras (Edmund Pixlink) were used. Doublets with focal lengths to ensure sufficient sampling and the desired field of view size were used in the imaging arm. The optical configuration chosen was tested using a grating with known periodicity and demonstrated to produce lateral spatial resolution as high as 0.3 μm and fields of view as large as 400 μm for all imaging modes. The imaging system also includes a high-load objective piezo-scanner (PI, P-726 PIFOC), with resolution of 0.3 nm and travel range of 100 μm to enable fine focussing and acquisition of axial image stacks. In practice a stack of images with different focus positions was obtained with each image being acquired within seconds of one another to account for any temperature related focal drifts over the time course of the experiment. The position in the image stack corresponding to the best focus was determined automatically by applying an entropy filter, with a 9 × 9 neighbourhood, to the acquired images and selecting the image with the highest mean entropy^21^. To enable long term monitoring of live cells a stage top electric incubator (Okolab) was also integrated into the instrument to maintain a humidified environment at a temperature of 37 °C and with an air flow consisting of 5% CO_2_. The operation of the CCD cameras, LEDs, objective scanner and SLM was controlled by an in-house written Labview interface.

### Quantitative phase contrast imaging

QPC imaging extends the traditional Zernike phase contrast microscope to a technique which is able to present topological images quantitatively. The basis of this approach can be understood by first considering an expression for the phase information in terms of intensity variation, used in interferometry:

1 $$I(x,y)=|{E}_{r}(x,y){|}^{2}+|{E}_{s}(x,y){|}^{2}+2{E}_{r}(x,y){E}_{s}(x,y)\cos [{\rm{\Delta }}\phi (x,y)]$$

where *x* and *y* are the local co-ordinates; *I* is the image intensity; *E*_*s*_ and *E*_*r*_ are the field distribution of the signal and the reference and *Δφ* is the phase difference between them. Zernike’s phase contrast microscope employs the same idea and the interference occurs between the background (*E*_0_) and the scattered light (*E*_1_). The image contrast arises from a π/2 phase shift and attenuation of the background light.

QPC imaging extracts the phase of the sample quantitatively by obtaining a set of images at equal phase intervals of δ, in this work δ is set to π/2. Here the intensity variation can be written as:

2 $$I(x,y;k)=|{E}_{0}(x,y){|}^{2}+|{E}_{1}(x,y){|}^{2}+2{E}_{0}(x,y){E}_{1}(x,y)\cos [{\rm{\Delta }}\phi (x,y)+k\delta ]$$

where k = 0, 1, 2, 3

The field amplitudes and the phase difference between the zero and diffracted orders can be retrieved as

3 $${\rm{\Delta }}\phi (x,y)=ta{n}^{-1}[\frac{I(x,y;3)-I(x,y;1)}{I(x,y;0)-I(x,y;2)}]$$

4 $$|{E}_{0}(x,y){|}^{2}+|{E}_{1}(x,y){|}^{2}=\sum _{k}I(x,y;k)/4$$

5 $${E}_{0}(x,y){E}_{1}(x,y)=\sqrt{{[I(x,y;3)-I(x,y;1)]}^{2}+{[I(x,y;0)-I(x,y;2)]}^{2}}$$

Note that the phase of (*x*, *y*) represents the phase between the diffracted order and zero order beam, it does not represent the phase delay of the beam propagating through the sample directly. The phase delay, (*x, y*), can be calculated from the parameters above using the below equation:

6 $$\theta (x,y)=ta{n}^{-1}[\frac{{E}_{1}(x,y)sin[{\rm{\Delta }}\phi (x,y)]}{{E}_{0}(x,y)+{E}_{1}(x,y)cos[{\rm{\Delta }}\phi (x,y)]}]$$

Equations 4 and 5 can be used to form a quadratic equation to solve for *E*_1_ and *E*_0_ whose ambiguity can be resolved by assuming (justifiably) that *E*_0_ > *E*_1_. Thus, the actual phase can be reconstructed and then converted to height using the material refractive index and wavelength of light.

### Image processing

**Figure 8:**
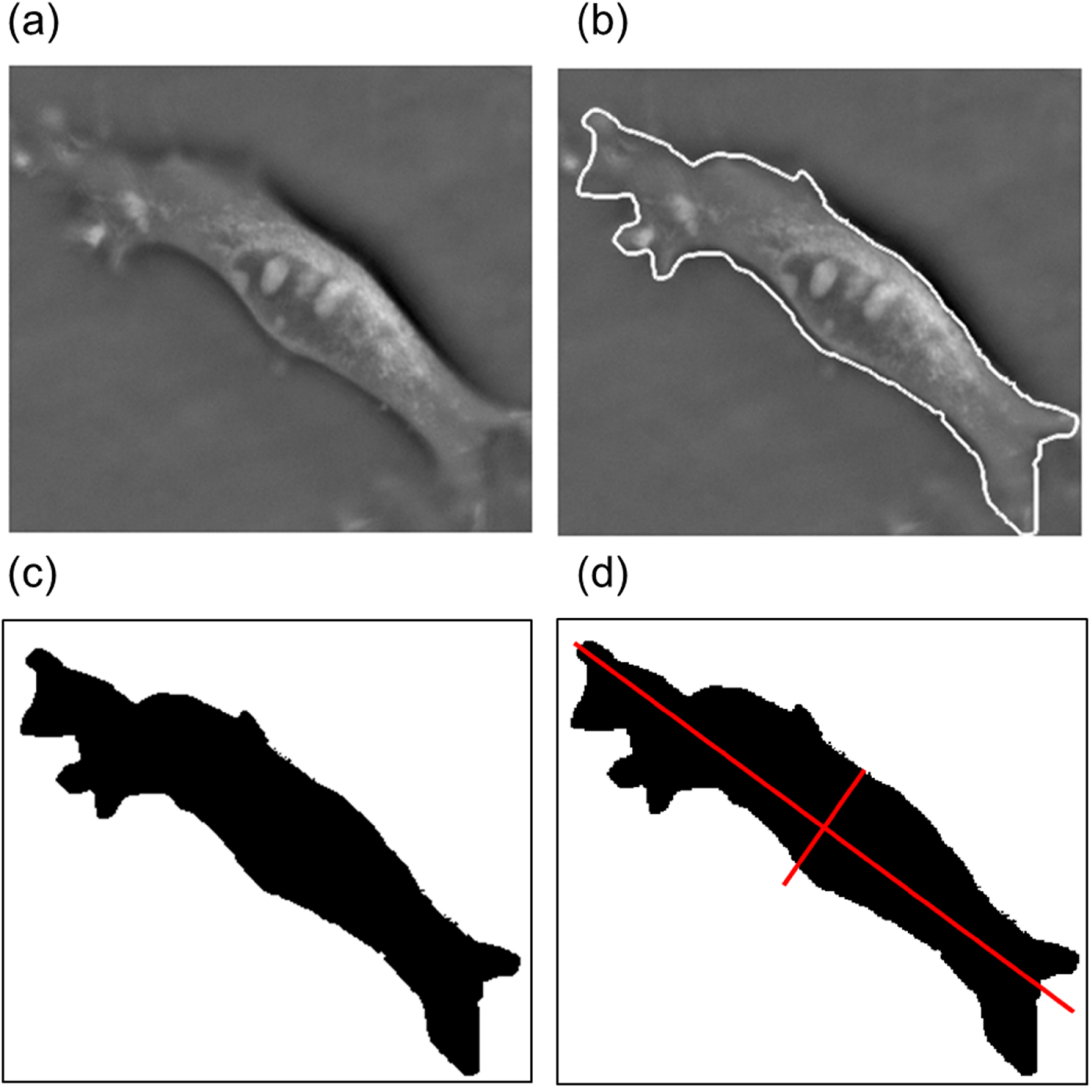
Image segmentation process: (**a**) Example cropped image of single cell; (**b**) manually traced outline of cell; (**c**) binary image of segmented cell; (**d**) major and minor axis overlayed on binary image as used for calculation of aspect ratio.

Quantitative assessment of cell size and morphology required the use of image processing techniques to segment the regions of interest corresponding to cells from the image background. At each time point as many as possible non-overlapping cells were selected from QPC and TIRM images and analysed to determine the cell area and aspect ratio, which denotes the ratio of the major axis length to the minor axis length of the cell. Cells to be analysed were first manually cropped and traced from the main image. Next the traced cells were segmented from the background via thresholding. Thersholding involved identifying the traced edges (via the use of a 3 × 3 Sobel edge filter), converting the resulting image to binary form and then filling the hole inside the traced area using the algorithm developed by Landini^22^. Binary forms of the cropped images were then analysed using inbuilt algorithms in Matlab (The Mathworks) to extract useful metrics. Here area was determined as the number of pixels belonging to each connected component identified as a cell. To calculate the length of the major and minor axes of the cell the best fitting ellipse to the region of interest was determined. A scalar specifying the length in pixels of the major axis and also the minor axis of the resulting ellipse that has the same normalized second central moments, for each axis, as the region was obtained. The aspect ratio was subsequently found from the ratio of the major axis length to the minor axis length. The steps involved in image processing are illustrated in Fig. 8.

### Cell Culture

C17.2 cells (used between passages 6–11) were obtained from the European Collection of Cell Cultures (ECACC) and maintained in DMEM + 2 mM Glutamine + 10% Foetal Bovine Serum (FBS). C17.2 cells were cultured to confluence in poly-L-lysine (10 µg/ml in sterile distilled water) pre-coated 75 cm^2^ flasks at 5% CO_2_, 37 °C. Once confluent, cells were detached from the flasks using 2.5% trypsin/EDTA and seeded in glass-bottom cell culture treated dishes (µ-Dish 35 mm, high, ibiTreat, tissue culture treated, sterile, 35 mm, 2 ml, Thistle Scientific) at a density of 1 × 10^5^. Cells were kept in 1.5 ml of DMEM:F12 medium supplemented with 1% N2, nerve growth factor (NGF) (200 ng/ml) and brain-derived neurotrophic factor (BDNF) (20 ng/ml) for 15 days with a medium change every 4 days.

### Immuno-staining

C17.2 cells were fixed in 4% paraformaldehyde for 20 minutes at room temperature and then permeabilized by incubating with Triton X-100 (0.1% v/v in PBS) for 10 minutes and blocked in 1% BSA/PBS for 30 minutes. Thereafter, BSA/PBS solution was aspirated and replaced with primary antibodies, diluted in 1% BSA/PBS. The primary antibodies used were: Mouse anti-nestin (Rat-401, 1 μg/ml, developed by S. Hockfield, obtained from DSHB, developed under auspices of NICHD, maintained by University of Iowa) (1:50); Rabbit anti-GFAP (Dako) (1:100); and Rabbit anti-α-βIII-tubulin (Cell Signalling Technology) (1:100). Cell samples were incubated with these primary antibodies for 2.5 hours. The primary antibody solution was then removed and cells washed with PBS (5 times). Secondary antibodies, Texas Red, anti-mouse IgG (Vector laboratories) (1:200); Texas Red, anti-rabbit IgG (Vector laboratories) (1:200); and Alexa flour 486 anti-rabbit IgG (Life Technologies) (1:400), were diluted according to manufacturer’s instructions in 1% BSA/PBS and then applied to the cells for 1 hour. The secondary antibody solution was then aspirated and cells washed with PBS extensively. Finally, cells were stained with 4′,6-diamidino-2-phenylindole dihydrochloride (DAPI).

## Electronic supplementary material

Supplementary Information
