# Supplementary material for: Label-Free, High Resolution, Multi-Modal Light Microscopy for Discrimination of Live Stem Cell Differentiation Status
Source: Sci Rep. 2018 Jan 15;8:697. doi: 10.1038/s41598-017-18714-y (PMC5768761; doi:10.1038/s41598-017-18714-y)
Supplement: Supplementary file 1 — Supplementary Information [file 41598_2017_18714_MOESM1_ESM.doc]

**SUPPLEMENTARY INFORMATION**

LABEL-FREE, HIGH RESOLUTION, MULTI-MODAL LIGHT MICROSCOPY FOR DISCRIMINATION OF LIVE STEM CELL DIFFERENTIATION STATUS

Jing Zhang^1^, Emilia Moradi^2^, Michael G Somekh^3^, Melissa L Mather^1,*^

^1^ Institute for Science and Technology in Medicine, Keele University, Stoke-on-Trent, ST4 7QB, United Kingdom

^2^ Optics and Photonics, Faculty of Engineering, University of Nottingham, Nottingham, NG7 2RD, United Kingdom

^3^ Department of Electronic and Information Engineering, The Hong Kong Polytechnic University, Hung Hom, Hong Kong,

^*^Correspondance to [m.mather@keele.ac.uk](mailto:m.mather@keele.ac.uk)

| Day of culture | **Number of cells** | | **Median data** | | | |
| --- | --- | --- | --- | --- | --- | --- |
|  | QPC | TIRM | Area QPC (pixels) | Area TIRM (pixels) | Aspect Ratio QPC | Aspect Ratio TIRM |
| 1 | 10 | 7 | 1.93x10^4^ | 1.71x10^4^ | 2.54 | 3.28 |
| 2 | 28 | 25 | 3.08x10^4^ | 2.97x10^4^ | 1.97 | 1.82 |
| 3 | 29 | 25 | 1.82x10^4^ | 2.79x10^4^ | 2.59 | 2.05 |
| 4 | 30 | 26 | 1.60x10^4^ | 2.31x10^4^ | 3.47 | 2.48 |
| 5 | 7 | 7 | 2.33x10^4^ | 1.80x10^4^ | 2.49 | 2.79 |
| 6 | 16 | 15 | 1.95x10^4^ | 6.84x10^4^ | 2.72 | 1.44 |
| 7 | 15 | 15 | 1.59x10^4^ | 6.56x10^4^ | 3.12 | 1.64 |
| 8 | 15 | 15 | 2.03x10^4^ | 6.36x10^4^ | 3.03 | 1.46 |
| 9 | 14 | 16 | 1.59x10^4^ | 7.20x10^4^ | 3.51 | 1.35 |
| 10 | 11 | 10 | 1.47x10^4^ | 7.92x10^4^ | 2.80 | 1.53 |
| 12 | 15 | 16 | 1.75x10^4^ | 8.80x10^4^ | 3.07 | 1.43 |
| 13 | 10 | 10 | 1.71x10^4^ | 9.45x10^4^ | 3.20 | 1.18 |
| 14 | 15 | 15 | 1.53x10^4^ | 9.53x10^4^ | 4.49 | 1.27 |
| 15 | 9 | 10 | 2.05x10^4^ | 1.52 x10^5^ | 2.93 | 1.30 |

Table 1: The number of cells analysed for each imaging modality and on each day of culture are displayed. Median values for the area and aspect ratio calculated on each day of culture and for each modality are also shown.

| Day of culture | **Area** | | | **Aspect Ratio** | | |
| --- | --- | --- | --- | --- | --- | --- |
|  | Rank sum | H value | p value | Rank sum | H value | p value |
| 1 | 96 | 0 | 0.60 | 79 | 0 | 0.31 |
| 2 | 776 | 0 | 0.73 | 816 | 0 | 0.29 |
| 3 | 689 | 0 | 0.061 | 884 | 0 | 0.14 |
| 4 | 187 | 0 | 0.062 | 259 | 0 | 0.28 |
| 5 | 54 | 0 | 0.90 | 50 | 0 | 0.80 |
| 6 | 136 | 1 | 0.0000023 | 339 | 1 | 0.0011 |
| 7 | 120 | 1 | 0.0000034 | 319 | 1 | 0.00036 |
| 8 | 141 | 1 | 0.00016 | 325 | 1 | 0.00014 |
| 9 | 105 | 1 | 0.0000036 | 313 | 1 | 0.000072 |
| 10 | 66 | 1 | 0.00012 | 164 | 1 | 0.0028 |
| 12 | 120 | 1 | 0.0000023 | 360 | 1 | 0.0000023 |
| 13 | 56 | 1 | 0.00025 | 146 | 1 | 0.0022 |
| 14 | 120 | 1 | 0.0000034 | 345 | 1 | 0.0000034 |
| 15 | 45 | 1 | 0.000022 | 116 | 1 | 0.035 |

Table 2: Results of Wilcoxon rank sum test displaying Rank sum, H value and p value for area and aspect ratio results on each day of culture. A H value of 1 corresponds to a rejection of the null hypothesis at a 5 % significance level indicating the two populations do not come from continuous distributions with equal medians.
